# Exploring the Role of Innate Lymphocytes in the Immune System of Bats and Virus-Host Interactions

**DOI:** 10.3390/v14010150

**Published:** 2022-01-14

**Authors:** Wan Rong Sia, Yichao Zheng, Fei Han, Shiwei Chen, Shaohua Ma, Lin-Fa Wang, Edwin Leeansyah

**Affiliations:** 1 Programme in Emerging Infectious Diseases, Duke-National University of Singapore Medical School, Singapore 169857, Singapore; wanrong.sia@duke-nus.edu.sg (W.R.S.); shiwei.chen@duke-nus.edu.sg (S.C.); linfa.wang@duke-nus.edu.sg (L.-F.W.); 2 Institute of Biopharmaceutical and Health Engineering, Tsinghua Shenzhen International Graduate School, and Precision Medicine and Healthcare Research Centre, Tsinghua-Berkeley Shenzhen Institute, Tsinghua University, Shenzhen 518055, China; zhengyc19@mails.tsinghua.edu.cn (Y.Z.); fayehan8023@sz.tsinghua.edu.cn (F.H.); ma.shaohua@sz.tsinghua.edu.cn (S.M.); 3 Center for Infectious Medicine, Department of Medicine, Karolinska Institutet, 14183 Stockholm, Sweden

**Keywords:** bat immunity, innate lymphocytes, innate-like T cells, viral pathogenesis

## Abstract

Bats are reservoirs of a large number of viruses of global public health significance, including the ancestral virus for severe acute respiratory syndrome coronavirus 2 (SARS-CoV-2) and the causative agent of coronavirus disease 2019 (COVID-19). Although bats are natural carriers of multiple pathogenic viruses, they rarely display signs of disease. Recent insights suggest that bats have a more balanced host defense and tolerance system to viral infections that may be linked to the evolutionary adaptation to powered flight. Therefore, a deeper understanding of bat immune system may provide intervention strategies to prevent zoonotic disease transmission and to identify new therapeutic targets. Similar to other eutherian mammals, bats have both innate and adaptive immune systems that have evolved to detect and respond to invading pathogens. Bridging these two systems are innate lymphocytes, which are highly abundant within circulation and barrier tissues. These cells share the characteristics of both innate and adaptive immune cells and are poised to mount rapid effector responses. They are ideally suited as the first line of defense against early stages of viral infections. Here, we will focus on the current knowledge of innate lymphocytes in bats, their function, and their potential role in host–pathogen interactions. Moreover, given that studies into bat immune systems are often hindered by a lack of bat-specific research tools, we will discuss strategies that may aid future research in bat immunity, including the potential use of organoid models to delineate the interplay between innate lymphocytes, bat viruses, and host tolerance.

## 1. Overview on Bat Immunity and Pathogen Tolerance

Bats are natural reservoirs [1,2,3,4,5,6,7] of many species of viruses, including coronaviruses [5], filoviruses [4,8], and paramyxoviruses [7,9], which cause severe morbidity during human spillover events [10]. However, such viruses rarely, if ever, cause any overt disease in bats. Accumulating evidence indicates that this is because bats use immune tolerance [8] as the primary response against viral infections instead of overt inflammatory responses. The host immune response to pathogens is traditionally described as a process of detection of non-self and elimination of the foreign materials. A less studied host defense response is tolerance [11,12], whereby the immune response is not primarily directed at pathogen clearance, but instead at limiting host tissue damage inflicted by the pathogens and to mobilize tissue repair mechanisms. Bats are highly diverse [13,14,15,16,17], and within this rich species diversity, certain traits including long lifespans [18], cancer resistance [19] and being viral reservoirs [2] are conserved. Notable gene families under intense selective pressure [17] include an expansion of DNA repair genes [17,20] and dampened inflammatory responses [21,22,23] originating from innate immune receptors [20,24]. These unique traits of bats are proposed to have co-evolved alongside powered flight and are reviewed in detail elsewhere [1,18,25,26].

Eutherian mammals’ innate and adaptive immune systems have evolved to sense pathogen infections and limit disease severity. Bridging these two systems and sharing the characteristics of both innate and adaptive immune cells are innate lymphocytes, which circulate in the periphery with the propensity to migrate and reside in non-lymphoid and mucosal barrier tissues [27,28,29]. They are highly abundant and are capable of mounting rapid effector immune responses. Therefore, innate lymphocytes serve as an ideal first line of defense against pathogen invasion, including those caused by viruses [27]. Although they may provide critical effector roles during viral infections, their overexuberant responses can also contribute to viral disease pathogenesis. Innate lymphocytes consist of highly diverse populations, including those that express germline-encoded antigen receptors, such as innate lymphoid cells (ILCs) and natural killer (NK) cells and those that express antigen receptors that undergo somatic recombination, including, but not limited to, mucosal-associated invariant T (MAIT) cells, natural killer T (NKT) cells, and γδ T cells. Innate lymphocytes that express specific antigen receptors are commonly known as innate-like T cells or unconventional T cells [27]. These cells express semi-invariant T cell receptors (TCRs) with restricted antigen receptor diversity. These receptors recognize a variety of antigenic structures; some subpopulations recognize peptide fragments as antigens presented by the classical major histocompatibility complex (MHC) proteins, and some other subsets recognize a variety of other antigenic structures presented by the non-classical MHC proteins. Additionally, these specialized immune cell populations can be indirectly activated through the actions of cytokines.

Until recently, a lack of available tools has hindered extensive studies into bat immune systems. Nevertheless, studies have shown the presence of these innate lymphocytes in various bat species [30,31,32] (Table 1). This mini-review will focus on the current knowledge of innate lymphocytes in bats, their function, and their potential role in antiviral immunity and tolerance. Despite the significant roles that γδT cells play in viral immunity and pathogenesis in humans, TCRγ- and TCRδ-related transcripts were found only in low abundance in bats [16]. Additionally, because there is no conclusive study on the presence and function of γδT cells conducted in bats, discussion of γδT cells in bat immunity will be the focus for future work. Finally, we will explore and discuss the potential use of organoid models to address the most pressing knowledge gap in bat immunological research, particularly in the interplay between innate lymphocytes and bat viruses and viral tolerance.

## 2. Innate Lymphoid Cells (ILCs)

ILCs are a heterogeneous group of cells belonging to the lymphoid lineage but do not possess recombined antigen receptors and lack lineage markers. ILCs are generally resident in non-lymphoid peripheral tissue, where these cells can quickly execute their effector functions upon receipt of the appropriate signals during infection or injury [28]. ILCs can be divided into five groups: natural killer (NK) cells, ILC1s, ILC2s, ILC3s, and lymphoid tissue-inducer (Lti) cells, with functionality mirroring that of conventional T cells. ILC1s, ILC2s, and ILC3s share similar properties to those of CD4+ T helper (Th) 1, Th2, and Th17 cells, respectively [41]. ILC1 and Th1 cells secrete IFNγ and TNF in response to intracellular pathogens and tumors; ILC2 and Th2 cells secrete IL-4, IL-5, IL-9, and IL-13 in response to large extracellular parasites; while ILC3 and Th17 cells secrete IL-17 and IL-22 in response to small extracellular pathogens. NK cells are similar to CD8+ T lymphocytes, and both are cytotoxic lineages, although the targeting mechanisms differ. Finally, LTi cells are generated early in embryonic development and instruct the formation of secondary lymphoid organs [41]. The effector function of the respective ILCs may be countered by the other ILC groups. ILCs mediate inflammation directly through MHC-II and indirectly through dendritic cells. ILCs are not only involved in the immune response against invading pathogens, but also in regulating thermogenesis, prevention of local inflammation, conversion of white fat to brown fat, and protection from high-fat diet-induced obesity and metabolic diseases [42].

In bats, ILCs have not been characterized. Given that group 2 ILCs favour a Th2 response [43], and that the Egyptian rousette bats were found to be the first mammalian species with more than one functional immunoglobulin E gene [44], the ILC2 group might be of interest for future bat research and in viral infection. High levels of IgE are usually associated with allergic inflammation [45] and coincide with certain viral infections [46], and the ILC2 group has been proposed to play a role in the initiation of allergic inflammation [43,47]. In patients with severe cases of COVID-19, circulating ILC2s were found to be depleted [48]. They may have homed to the inflammatory sites, as ILC2s are important in mediating the process of tissue repair by the secretion of cytokines and other factors involved in wound healing and tissue remodeling [49]. The secreted factors stimulate the proliferation and differentiation of epithelial cells in conditions as diverse as helminth and viral infections.

## 3. Natural Killer (NK) Cells

NK cells play a role in immune defenses against viral infections [39] and tumorigenesis [33,50]. Using a diverse set of activating and inhibitory receptors that recognize MHC-I, cell surface receptors, and crystallizable fragment (Fc) domain of antibodies, activated NK cells secrete cytolytic proteins and inflammatory cytokines [29]. In humans, NK cells express highly polymorphic killer-cell immunoglobulin-like receptors (KIRs) and the C-type lectin receptors (KLRs) heterodimer, which bind to HLA class I molecules, and their combination results in a spectrum of different reactivity [38]. Virus-infected and aberrant cells often have dysregulated MHC-I expression, causing loss of inhibitory NK state through KIR and CD94/NKG2A interaction, resulting in NK cell activation [29,38]. In addition, there are also subsets of NK cells, such as the uterine NK cells, which play a more immune suppressive and tolerance role [51].

Previous studies have revealed unique features of MHC-I genes [20,52], a less restrictive MHC-I peptide binding groove [53], and inhibition or loss of NK receptor families in bats [16,20]. Moreover, NK cells in bats seem to have undergone unique negative regulation [16,17,20,31]. Papenfuss noted the lack of KIRs in the fruit-eating bat *Pteropus alecto* transcriptomic dataset [16], which is in concordance with other independent studies on the absence of KIRs in *P. alecto* and *Myotis davidii*, *Rousette aegyptiacus*, and finally, across the order Chiroptera represented by 28 bat species in the most recent study by Moreno [20]. Given that the MHC-I gene family seems to have expanded outside the canonical MHC-I region [31] and is lacking in alpha and kappa blocks [52] in bats, does this loss of KIR genes then prompt a less activating state as a result of stress from flight and infection? Moreover, KIR+ NK cells are associated with a more mature and cytolytic NK cell state [54], which may not be ideal for bats, as it may promote a state of broken tolerance. In humans, certain KIR expressions have been found to be associated with different disease outcomes in virus infections, such as SARS-CoV-2 [55], SARS-CoV [56], influenza [57], and HIV [38]. In addition, NKG2-like genes such as inhibitory NKG2A and activating NKG2D [16,20,31] were detected in multiple species of bats. These receptors with inhibitory signaling motifs were found to be conserved at higher transcript levels, suggesting a favored inhibitory NK cell state.

## 4. Mucosal-Associated Invariant T Cells

Mucosal-associated invariant T cells (MAIT cells) are a population of unconventional, innate-like T cells that were initially discovered in the intestinal lamina propria of mice [58]. In humans, MAIT cells are defined by the expression of the TCR Vα7.2 segment joined with the Jα12/20/33 segment and coupled with restricted TCR Vβ segments, primarily Vβ2 and Vβ13 [59]. Mature human MAIT cells are predominantly CD8+ and express high levels of the lectin-like receptor CD161. In humans, MAIT cells represent 5–10% of circulating T cells in the blood and are abundant in tissues, including the lungs, intestines, and the liver [60]. The MAIT cell TCR recognizes microbial-derived riboflavin-related metabolic intermediates and some structurally-related non-microbial antigens presented on the evolutionarily conserved MHC-Ib-related protein (MR1) [61,62,63]. The high similarity of MR1 molecules across eutherian mammals [30,64] likely signifies the important role of MR1 and MAIT cells and other MR1-restricted T cells throughout evolution [65,66]. Besides sensing bacteria-infected cells, MAIT cells can be activated independently of their TCR through inflammasome-derived and innate cytokines, including IL-1β, IL-12, IL-18, and type I interferon [67]. MAIT cells play important and often protective roles in various bacterial infectious diseases in human and animal models [68]. MAIT cells are also associated with several viral infections such as influenza [69,70], HIV-1 [71,72], and SARS-CoV-2 [73,74,75,76,77,78,79].

In recent studies, the use of MR1 tetramers loaded with MAIT cell antigens have facilitated a more specific identification of this cell type. Using this technology, we have identified MR1-restricted T (MR1T) cells in the fruit-eating bat *Pteropus alecto* [30,80]. As observed in humans, these MR1T cells represent a significant population at baseline. However, unlike human MR1-restricted MAIT cells, *P. alecto* MR1T cells are not capable of cytokine production without prior antigenic priming [30]. Interestingly, the use of recombinant human IL-2 and IL-7 were able to prime and support *P. alecto* MR1T cell proliferation. When stimulated with the MR1 ligand 5-OP-RU and riboflavin-synthesis competent bacteria, primed *P. alecto* MR1T cells produce perforin, TNF, and IL-17 as detected by flow cytometry. Using cross-reactive antibodies, we detected the expression of MAIT cell-associated transcription factors PLZF, RORγt, T-bet, and Eomes in *P. alecto* MR1T cells. Similar to human MAIT cells, primed *P. alecto* MR1T cells can kill cells fed with riboflavin synthesis-competent bacteria and the MR1 ligand 5-OP-RU. This cytotoxic ability of *P. alecto* MR1T cells is equally preserved against target cells of *P. alecto* and human origin, underscoring the high conservation between human and bat MR1. Whether these *P. alecto* MR1T cells are restricted by a semi-invariant TCR sequence has yet to be determined.

Intriguingly, in SARS-CoV-2 infection, several groups have reported a significant depletion of MAIT cells in the circulation, which have likely homed to the infected and inflamed lung tissues. Indeed, excessive activation of MAIT cells in COVID-19 exacerbates the disease [74,76,78] and is an independent predictor of death [74]. Because activated MAIT cells are highly cytotoxic and are a potent source of proinflammatory cytokines in the lungs and intestines, MAIT cells may thus amplify the excessive inflammation seen in COVID-19 disease through the activation and recruitment of other immune cells in mucosal tissues. On the other hand, MAIT cells have a potent antiviral capacity [81,82] that may initially limit viral replication during the early stages of infection. How would bats then be able to minimize disease severity if MR1T cells are a considerable population in vivo? In our previous study, we noted that the *P. alecto* MR1T cells resolved their inflammatory responses within 24 h [30], whereas human MAIT cells continued to display inflammatory responses days after the initial stimulation [83]. Whether this is due to the role of anti-inflammatory factors secreted by other cell types within the in vitro culture system or due to certain mechanistic regulations intrinsic to the MR1T cells would be interesting to address in future studies.

## 5. Invariant Natural Killer T (iNKT) Cells

Another small subset of innate-like T cells implicated in viral pathogenesis in humans is the invariant natural killer T (iNKT) cells. This cell type is restricted by the MHC-I-like molecule CD1d and can be activated by CD1d-presented endogenous and pathogen-derived glycolipid antigens. The presence of iNKT cells in bats has not been directly shown, but the presence of putative CD1d sequences in *P. alecto* [16] suggests that populations of CD1d-restricted T cells, including iNKT-like cells, may exist in bats. How would the putative CD1d-restricted T cells contribute to bat antiviral immunity? While there are no known lipid antigens of viruses that would be presented by the CD1d molecule, the cytokine milieu generated during virus infection is able to activate iNKT cells in mouse models and in humans [84]. Furthermore, certain viral infections alter the presentation of endogenous lipid antigens, leading to the activation of iNKT cells and the elimination of infected cells [84,85]. It remains to be explored if bats could express endogenous lipid antigens that may activate CD1d-restricted T cells in a similar fashion.

## 6. Future Research: The Use of Organoids to Decode Bat Innate Immune Responses to Viral Infections

The tools commonly used to establish a living model to understand bat immunology are cell lines, organoids, and bat colonies. Nevertheless, establishing a distinct cell line of bat origins is technically challenging and laborious. Furthermore, a single cell type may not be sufficient to propagate a plethora of viruses, and a model based on one certain cell line cannot model the complex structure and cell interactions in original tissues [26,86,87]. Compared with cell lines, captive breeding of colonies of bats allows comprehensive analysis of bat immune systems with high reliability [26,87]. However, it is difficult to establish a bat colony for some species. A captive breeding colony of *Rinolophus sinicus*, for example, the potential reservoir of SARS-CoV-2, has not been established so far [87]. Another unique approach to studying bat immunity would be to develop a chimeric bat–mouse model, which has been demonstrated in *Eonycteris spelaea* [88]. In this model, the bat immune cells transplanted to immunodeficient mice could survive, expand, and repopulate in the recipients. Thus, the platform recapitulates the bat immune system in mice without causing obvious abnormalities [88]. However, the impacts of mouse physiology on bat immune cells are unclear. It also remains to be seen whether this approach can be uniformly applied to different bat species. Further advancements in bat immunity research have been achieved by improving sequencing technologies and capabilities, as well as by the development of bat-specific research tools such as antibodies against cell surface markers [26,87]. In vitro studies such as the use of cell lines and bat organoids may further aid in the understanding of how bats deal with viral infections and remain largely disease-free [26,87,89]. The feasibility of using organoids to understand virus–host interactions has also been demonstrated by several groups [89,90].

Organoids are miniaturized and simplified three-dimensional (3D) models of organs, ranging in size from micrometers to millimeters and growing from embryonic stem cells, induced pluripotent stem cells (iPSCs), neonatal tissue stem cells, or adult progenitor cells [91]. These cells can divide and differentiate into various subsets and self-organize to form a tissue-like mass which resembles the structures and functions of its counterpart organ. To date, different types of organoids have been created fulfilling a variety of research purposes and clinical applications [91,92,93]. For instance, tonsil organoids modeling key germinal center features were recently generated to investigate the mechanisms of human adaptive immune response in vitro and to evaluate the effectiveness of vaccine candidates in an entirely human system [94]. In addition, human intestinal organoids containing lamina propria-derived CD4+ T cells were developed to reveal the impacts of intestinal stem cell–immune cell interactions in fetal intestine development and inflammation [95]. More recently, a lung organoid-on-a-chip system was manufactured to study SARS-CoV-2-induced aberrant immune responses that lead to lung injury, and it was further used to test for antiviral drugs [96]. The unique structures and complex components of organoids facilitate a comprehensive analysis of multiple cells interactions that are otherwise hard to recapitulate in traditional two-dimensional (2D) cell cultures. As bat organoids are established by using bat tissues, it is expected to recapitulate the genetic features of the bats of origin. In addition, generating, maintaining, passaging, and replicating organoids is relatively easier than establishing the captive breeding colonies of bats, partially due to their low fecundity [1]. Thus, organoids may facilitate etiological studies and potential drug development in vitro [91,92,93]. However, it remains to be seen if the methods used to generate human or mouse organoids can be directly applied to the generation of bat organoids. Nevertheless, bat intestinal organoids were recently developed to study bat-borne viral infections and bat immunity [87,89].

Novel therapeutic strategies against inflammatory or infectious diseases might be developed by understanding the innate immunity of bats. In this respect, bat enteroids with a multicellular composition of intestinal epithelium have been successfully developed from horseshoe bat species *Rhinolophus sinicus* to recover SARS-CoV-2 replication in vitro [89]. It suggests that bat organoids are effective tools to propagate a variety of bat-borne viruses that are otherwise difficult to culture [86]. This is probably due to tissue tropism of bat virus and innate immune tolerance mechanisms conferred to bat organoids. Only bat intestinal organoids have been successfully developed to date [89]. The interest in choosing bat gut tissues as a resource of bat organoid culture probably arose because many bat-borne coronaviruses were found in samples of bat gut origin [97,98]. It will be interesting to study the virus infection of different bat tissues using organoids. For instance, establishing bat lung organoids may help us understand bat defense strategies against respiratory viral infections, thus shedding light on developing novel treatments of SARS-CoV-2. Interestingly, bat induced pluripotent stem cells (iPSCs) were generated by using somatic reprogramming protocols [99,100]. Going forward, these techniques may facilitate the generation of multiple types of bat organoids that allow for a comprehensive investigation of various viral infections in different tissues, while reducing the need to generate the bat organoids from the tissues of captured wild bats or those from breeding colonies. Alternatively, bat embryonic stem cells could also be a potential resource for generating different types of bat organoids.

**Figure 1:**
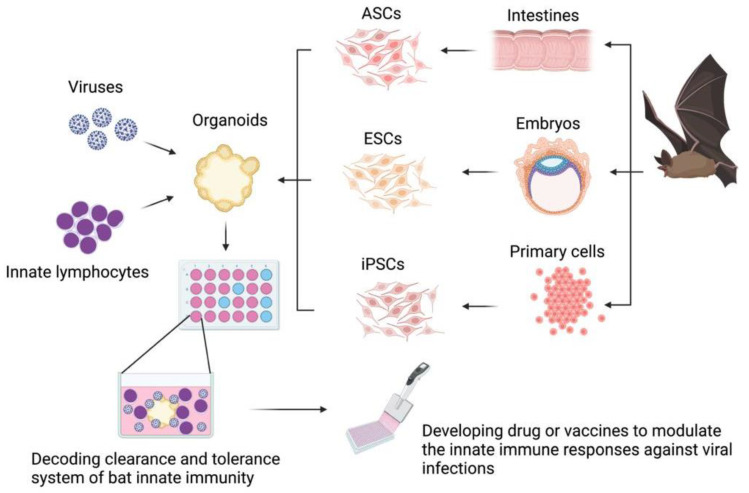
Generation and potential application of organoid platforms in immunological studies in bats. Bat organoids could be generated from adult stem cells or progenitor cells of normal tissues, embryonic stem cells of embryos, or induced pluripotent stem cells reprogrammed from somatic primary cells. Bat organoids cocultured with their own innate lymphocytes and challenged with bat-borne viruses could be used to study tolerance and clearance mechanisms of bat innate immune systems. Decoding bat innate immune systems is expected to assist in drug development and vaccine design. The figure was created in BioRender.com (www.biorender.com, accessed on 6 December 2021). Abbreviations: ASCs, adult stem cells; ESCs, embryonic stem cells; iPSCs, induced pluripotent stem cells.

The bat organoid platform is expected to model bat immunity and study the interactions of immune cells and bat viruses, including the lymphocyte activation and function augmentation upon virus infection, as well as the capacity of innate lymphocytes in restricting viral replication (Figure 1). Though the use of organoids for bat immunology research is still in its infancy, modern techniques can facilitate the development and use of the organoid modeling capacity. For instance, quantitative RT-PCR and single-cell RNA sequencing (scRNA-seq) techniques allow for comprehensive analysis of cellular and molecular responses of bat organoids to various virus infections. Omics, including both transcriptomics and proteomics, of bat organoids might serve as routine measures to study innate immunity in bats and their interplay with bat viruses [1,26,87].

In addition, as antibodies against surface markers of bat immune cells were recently validated [101,102,103], they could be used to identify the immune cell compositions of bat organoids and to isolate the bat immune cells from bat organoids, thus allowing for the study of immune responses of innate lymphocytes toward virus infections. Furthermore, because an automated organoid platform and a machine learning algorithm have been recently established [104,105], the combination of organoid manufacturing technology and the learning-based assessment system may potentially allow a high throughput modulation and analysis of the innate immune response of bats to virus infections in the future.

## 7. Concluding Remarks

In summary, the emerging data on innate lymphocytes in bats suggest that these unique immune cell populations may play an important role in controlling viral infections in bats. The recent successful establishment of bat intestinal organoids for SARS-CoV-2 infection and advances in organoid engineering promise to be an appealing tool to address the most pressing knowledge gaps in bat immunological studies, including the need to identify, culture, and investigate the different functions of specific immune cell populations in various bat tissues derived from multiple bat species, as well as the interplay between bat immune cells with various bat viruses. Knowledge gained from such immunological studies may help us understand how bats deal with viral infections and remain largely disease-free.

## Figures and Tables

**Table 1:** Brief summary of known characteristics of innate lymphocytes in humans and bats.

Innate Lymphocytes in Bats	Innate Lymphocytes in Humans
NK cells	
CD3^−^Tbet^+^Eomes^+^ cell population could be regarded as NK cells in *P. alecto* bats; transcripts of CD56 and CD16 genes present [16].	Human NK cells are identified as CD56^+^CD3^−^ and are functionally heterogeneous based on differential expressions of CD56 and CD16 [33,34,35]. Circulating NK cells constitutively express T-bet, EOMES, IL-2 and IL-15 receptor [36].
Frequency and location of NK cells in bats currently unknown.	NK cells represent 7–25% of all circulating lymphocytes in humans and are abundant in tissues with different phenotypes [37].
Absence of KIR genes across bats species. Inhibitory NKG2A/CD94 and activating NKG2D found. However, ligand and downstream signaling protein for NKG2D appears to be absent [20].	Express a diverse set of activating and inhibitory receptors, such as KIR and KLR receptors [29,38].
Function unknown.	NK cells have a role in controlling viral infections and prevent tumorigenesis [33,39].
NKT cells	
Surface markers unknown.	Express both T cell and NK cell markers.
CD1d transcripts detected in *P. alecto* [16]. TCR segment usage is unknown.	Type I NKT cells recognize glycolipid antigens presented by CD1d [40]. Express TCR Vα24-Jα18 with limited TCR Vβ repertoires (predominantly Vβ11).
No information related to function of NKT cells in bats.	NKT cells are involved in both bacterial and viral infections.
MAIT cells	
*P. alecto* MR1T cells are defined as hMR1-5OP-RU tetramer+ and intracellular CD3^+^ cells [30]. TCR segment usage is unknown.	Canonical MAIT cells are defined as Va7.2^+^ CD161^+^hMR1-5-OP-RU tetramer^+^ CD3^+^ cells. Express TCR Vα7.2-Jα12/20/30 with limited TCR Vβ repertoires (predominantly Vβ2 and Vβ13).
*P. alecto* MR1T cells may comprise 30% of CD3^+^ T cells compartments in the peripheral blood [30].	Abundance of MAIT cells in blood; 5–10% of total T cells [27].
*P. alecto* MR1T cells recognize MR1 molecule [30].	MAIT cells recognize MR1 molecule.
Antibacterial capacity demonstrated in *P. alecto*. Antiviral function is unknown.	Antibacterial and indirect antiviral role demonstrated.
Other innate lymphocytes: ILCs/γδT cells/Lti cells in bats remain to be explored.	

Abbreviations: NK cells, natural killer cells; T-bet, T-box expressed in T cells; EOMES, eomesodermin; IL, interleukin; NKG2, natural killer group 2; KIR, killer Ig-like Receptor; KLR, killer lectin-like receptor; NKT, natural killer T; TCR, T cell receptor; MAIT cells, mucosal associated invariant T cells; MR1, MHC-Ib protein; 5-OP-RU, 5-(2-oxopropylideneamino)-6-D-ribitylaminouracil; ILCs, innate lymphoid cells; γδT cell, gamma delta T cell, Lti cells, Lymphoid tissue inducer cells.

## Data Availability

Not applicable.
